# Integration of a Cultural Complications Curriculum Into a Surgery Department Conference

**DOI:** 10.1001/jamanetworkopen.2025.17811

**Published:** 2025-06-27

**Authors:** Elise E. H. Fannon, Rachel Ekaireb, Alexandra Johns, Priya Suri, Diana Farmer, Gregory Jurkovich, Luis Godoy, Kathryn M. Stadeli

**Affiliations:** 1 Department of Surgery, University of California, Davis Medical Center, Sacramento; 2 Department of Surgery, David Grant Medical Center, Travis Air Force Base, California

## Abstract

This survey study assesses outcomes and perceptions of a cultural complications curriculum implemented at a Department of Surgery Morbidity and Mortality conference.

## Introduction

Disparities in surgical outcomes persist among disadvantaged patient populations due to racial, ethnic, and socioeconomic inequities. Cultural competency is critical in mitigating these disparities, yet formalized education remains inconsistent across surgical training programs. Our study evaluates the outcomes of a cultural complications (CC) curriculum on attitudes, knowledge, and behaviors related to cultural competency implemented within the University of California Davis Department of Surgery Morbidity and Mortality (MM) Conference.

## Methods

A single-institution, prospective interventional survey study was conducted from February 2022 to August 2023, using anonymous, voluntary surveys of University of California Davis Department of Surgery members to assess attitudes, knowledge, and behaviors related to CC. The study followed the AAPOR reporting guideline and was determined exempt from review and the requirement of informed consent by the University of California Davis institutional review board. The CC curriculum consisted of 4 case-based discussions integrated into MM conferences. Cases were anonymously submitted, ensuring relevance to clinical challenges. Participants attended the following CC cases: (1) patient-clinician racial bias (9 postcurriculum respondents), (2) obesity stigma (9 postcurriculum respondents), (3) limited English proficiency (15 postcurriculum respondents), and (4) clinician bias toward patients with a history of substance use (27 postcurriculum respondents). Overall, 12 respondents attended 1 session, 9 respondents attended 2 sessions, 6 respondents attended 3 sessions, and 3 respondents attended all 4 sessions. Demographic information (age, gender, identifying as LGBTQAI, race [Asian, Black, White, and other (American Indian and Alaska Native, Native Hawaiian or Pacific Islander, and any race not otherwise specified)], and ethnicity [Hispanic and non-Hispanic]) was collected as part of the survey.

Precurriculum and postcurriculum Likert scale surveys (eMethods in Supplement 1) assessed participants’ perceptions of CC frequency, their comfort in managing CCs, and the adequacy of existing MM discussions on the topic. Responses ranged from 1 (strongly disagree or never) to 5 (strongly agree or always), with higher scores indicating greater cultural competency awareness and engagement. The surveys were anonymous, with no linkage between preresponses and postresponses. Data were analyzed using unpaired *t* tests to measure statistical significance at a confidence level of 95% (ie, *P* < .05). Data were analyzed October 2023 to December 2024 using Intercooled Stata 8.0 (StataCorp).

## Results

**Figure 1.**
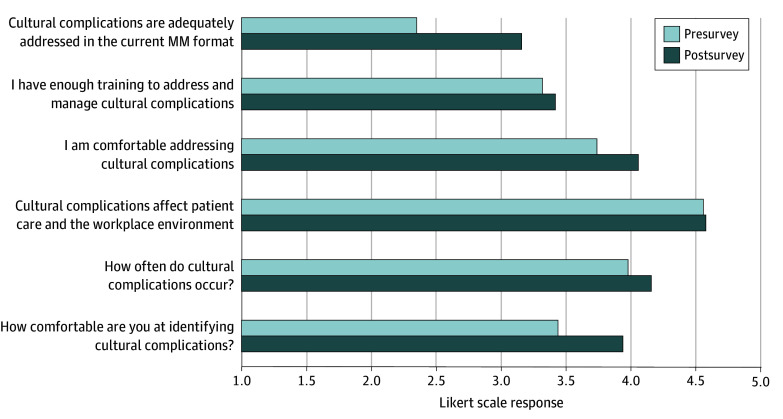
Outcomes of the University of California Davis Department of Surgery Morbidity and Mortality (MM) Cultural Complications Curriculum The graph reflects the mean Likert scale response to each survey question. Responses range from 1 (strongly disagree or never) to 5 (strongly agree or always).

**Figure 2.**
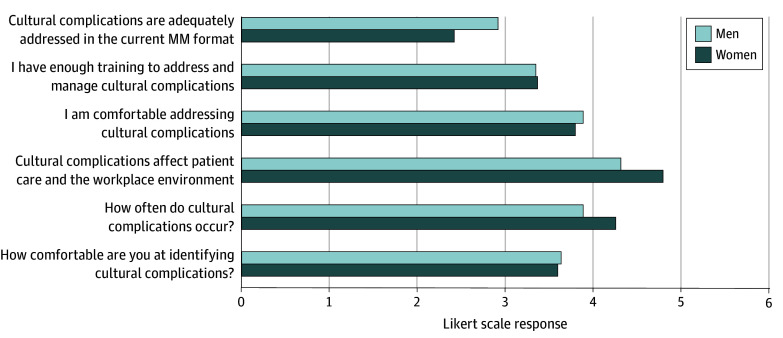
Perceptions of Cultural Complications Curriculum by Self-Identified Gender The graph reflects the mean Likert scale response to each survey question. Responses range from 1 (strongly disagree or never) to 5 (strongly agree or always). MM indicates morbidity and mortality.

There were 43 precurriculum responses and 31 postcurriculum responses, representing a diverse cohort, primarily aged 25 to 44 years (precurriculum: 26 respondents [60.5%]; postcurriculum : 23 respondents [74.2%]), with most identifying as White (precurriculum: 23 respondents [53.5%]; postcurriculum: 15 respondents [48.4%]) and non-Hispanic (precurriculum: 39 respondents [90.7%]; postcurriculum: 27 respondents [87.1%]). There were more women who responded precurriculum (23 respondents [53.5%]) and more men who responded postcurriculum (18 respondents [58.1%]). Smaller proportions identified as Asian (16 of 74 respondents [21.5%]), Black (8 of 74 respondents [10.8%]), LGBTQAI (12 of 74 respondents [16.2%]), or other underrepresented groups. Survey responses indicated that both precurriculum and postcurriculum participants believed that CCs occurred often (mean [SD] response, 4.0 [1.1] vs 4.2 [1.0]) and strongly agreed that CCs affect clinical care (mean [SD] response, 4.6 [0.9] vs 4.6 [0.9]) (Figure 1). Postcurriculum results demonstrated increased recognition of CCs (mean [SD] response, 3.4 [1.2] vs 3.9 [0.8]; *P* = .046; mean difference, −0.49; 95% CI,−0.98 to −0.01) and reported that CCs were more adequately addressed in MM (mean [SD] response, 2.4 [0.9] vs 3.2 [1.3]; *P* = .002; mean difference, −0.81; 95% CI, −1.31 to −0.31). However, there was no significant change in improved comfort addressing them (mean [SD] response, 3.8 [0.9] vs 4.1 [0.6]) or perceived sufficiency of training to manage CCs (mean [SD] response, 3.3 [1.0] vs 3.4 [0.9]). Gender subgroup analysis found that men were less likely than women to acknowledge the frequency and impact of CCs (Figure 2).

## Discussion

This survey study found that the integration of a CC curriculum into surgical MM conferences was associated with enhanced awareness and discussion of sociocultural factors impacting patient care. These findings align with prior studies demonstrating that structured, case-based CC training improves clinical awareness and decision-making.^1,2^ While participants reported greater recognition and engagement with CCs, persistent gaps highlight the need for continued efforts, including longitudinal training and institutional commitment.^3,4^ An institutional change resulted from the curriculum: a new category—cultural, racial, gender, and socioeconomic factors—was added to the department’s root-cause analysis diagram to encourage systematic consideration of these elements in surgical complications.

Gender-based differences in CC perceptions emphasize the need for inclusive interventions. Implicit biases may influence how clinicians perceive and address CCs, reinforcing the value of curricula that challenge assumptions and promote equity.^5,6^

This study is subject to limitations, including selection bias, reliance on self-assessment, and a low response rate, which may affect generalizability. Future research should explore long-term outcomes and clinician behavior. Moving forward, institutions should embed cultural competency in surgical training and consider using MM conferences as a lasting platform for growth.

## Notes

Supplement 1. **eMethods.** Precurriculum and Postcurriculum Questionnaires

Supplement 2.
Data Sharing Statement
