# Supplementary material for: Integration of a Cultural Complications Curriculum Into a Surgery Department Conference
Source: JAMA Netw Open. 2025 Jun 27;8(6):e2517811. doi: 10.1001/jamanetworkopen.2025.17811 (PMC12205394; doi:10.1001/jamanetworkopen.2025.17811)
Supplement: Supplement 1. — eMethods. Precurriculum and Postcurriculum Questionnaires [file jamanetwopen-e2517811-s001.pdf]

Supplemental Online Content

Fannon EEH, Ekaireb R, Johns A. Integration of a cultural complications curriculum into a surgery department conference. *JAMA Netw. Open*. 2025;8(6):e2517811.
doi:10.1001/jamanetworkopen.2025.17811

eMethods. Presurvey and Postsurvey Questionnaires

This supplemental material has been provided by the authors to give readers additional information about their work.

© 2025 Fannon EEH et al. *JAMA Network Open*

eMethods. Presurvey and Postsurvey Questionnaires

Cultural Complications Pre-Survey

Start of Block: Demographics

Gender What is your gender?

○ Male (1)

○ Female (2)

○ Non-binary / third gender (3)

○ Prefer not to say (4)

Age What is your age?

○ Under 18 (1)

○ 18 - 24 (2)

○ 25 - 34 (3)

○ 35 - 44 (4)

○ 45 - 54 (5)

○ 55+ (6)

Ethnicity1 Are you of Hispanic, Latino, or Spanish origin?

- Yes (2)
- No (1)

Ethnicity2 How would you describe yourself? Please select all that apply.

- White (1)
- Black or African American (2)
- American Indian or Alaska Native (3)
- Asian (4)
- Native Hawaiian or Pacific Islander (5)
- Other (6)

Q9 Do you identify as part of the LGBTQI+ community?

- No (1)
- Yes (2)

Page Break

© 2025 Fannon EEH et al. *JAMA Network Open*

Q10 How comfortable are you at identifying cultural complications when they occur in the workplace setting?

- Not comfortable at all (1)
- Slightly comfortable (2)
- Moderately comfortable (3)
- Very comfortable (5)
- Very Comfortable (5)

Q11 How often do cultural complications occur in the hospital/clinic/education setting?

- Almost never (1)
- Few times a year (2)
- Monthly (3)
- Weekly (4)
- Daily (5)

Q12 Cultural complications affect patient care and the workplace environment.

- Strongly disagree (1)
- Somewhat disagree (2)
- Neither agree nor disagree (3)
- Somewhat agree (4)
- Strongly agree (5)

Q13 I am comfortable addressing cultural complications in the hospital/clinic setting.

- Strongly disagree (1)
- Somewhat disagree (2)
- Neither agree nor disagree (3)
- Somewhat agree (4)
- Strongly agree (5)

Q14 I have enough training to address and manage cultural complications between co-workers, staff, and patients.

- Strongly disagree (1)
- Somewhat disagree (2)
- Neither agree nor disagree (3)
- Somewhat agree (4)
- Strongly agree (5)

© 2025 Fannon EEH et al. *JAMA Network Open*

Q15 Cultural complications are adequately addressed in the current general surgery M&M format?

- Strongly disagree (1)
- Somewhat disagree (2)
- Neither agree nor disagree (3)
- Somewhat agree (4)
- Strongly agree (5)

Page Break

© 2025 Fannon EEH et al. *JAMA Network Open*

End of Block: Demographics

Start of Block: Block 1

Q27 Do you have any concepts you hope are addressed in these cultural complications discussions?

____

End of Block: Block 1

© 2025 Fannon EEH et al. *JAMA Network Open*

Cultural Complications Post-Survey

Start of Block: Default Question Block

. Thank you for answering some follow-up questions to assess the impact of the UC Davis Cultural Complications M&M Curriculum. All responses are anonymous and greatly appreciated.

End of Block: Default Question Block

Start of Block: Demographics

Q1 What is your gender?

- ◯ Male (1)
- ◯ Female (2)
- ◯ Non-binary / third gender (3)
- ◯ Prefer not to say (4)

Q2 What is your age?

- ◯ Under 18 (1)
- ◯ 18 - 24 (2)
- ◯ 25 - 34 (3)
- ◯ 35 - 44 (4)
- ◯ 45 - 54 (5)
- ◯ 55+ (6)

© 2025 Fannon EEH et al. *JAMA Network Open*

Q3 Are you of Hispanic, Latino, or Spanish origin?

- ○ Yes (2)
- ○ No (1)

Q4 How would you describe yourself? Please select all that apply.

- ☐ White (1)
- ☐ Black or African American (2)
- ☐ American Indian or Alaska Native (3)
- ☐ Asian (4)
- ☐ Native Hawaiian or Pacific Islander (5)
- ☐ Other (6)

Q5 Do you identify as part of the LGBTQI+ community?

- ○ No (1)
- ○ Yes (2)

Page Break

© 2025 Fannon EEH et al. *JAMA Network Open*

Q6 How comfortable are you at identifying cultural complications when they occur in the workplace setting?

- Not comfortable at all (1)
- Slightly comfortable (2)
- Moderately comfortable (3)
- Somewhat comfortable (4)
- Very Comfortable (5)

Q7 How often do cultural complications occur in the hospital/clinic/education setting?

- Almost never (1)
- Few times a year (2)
- Monthly (3)
- Weekly (4)
- Daily (5)

© 2025 Fannon EEH et al. *JAMA Network Open*

Q8 Cultural complications affect patient care and the workplace environment.

- Strongly disagree (1)
- Somewhat disagree (2)
- Neither agree nor disagree (3)
- Somewhat agree (4)
- Strongly agree (5)

Q9 I am comfortable addressing cultural complications in the hospital/clinic setting.

- Strongly disagree (1)
- Somewhat disagree (2)
- Neither agree nor disagree (3)
- Somewhat agree (4)
- Strongly agree (5)

Q10 I have enough training to address and manage cultural complications between co-workers, staff, and patients.

- Strongly disagree (1)
- Somewhat disagree (2)
- Neither agree nor disagree (3)
- Somewhat agree (4)
- Strongly agree (5)

Q11 Cultural complications are adequately addressed in the current general surgery M&M format.

- ○ Strongly disagree (1)
- ○ Somewhat disagree (2)
- ○ Neither agree nor disagree (3)
- ○ Somewhat agree (4)
- ○ Strongly agree (5)

Page Break

© 2025 Fannon EEH et al. *JAMA Network Open*

Q16 I enjoyed participating in the cultural complications M&M presentations over the past year.

- Strongly disagree (1)
- Somewhat disagree (2)
- Neither agree nor disagree (3)
- Somewhat agree (4)
- Strongly agree (5)

Q17 Cultural complications M&Ms were effective at increasing my understanding of cultural complications and how they can impact our patients and care teams.

- Strongly disagree (1)
- Somewhat disagree (2)
- Neither agree nor disagree (3)
- Somewhat agree (4)
- Strongly agree (5)

End of Block: Demographics

Start of Block: Block 2

Q13 Which of the following Cultural Complications Presentations did you attend (Please select all that apply)?

- ☐ Patient request for provider of different race (initial GR presentation) (1)
- ☐ Obesity stigma (2)
- ☐ Care for Limited English Proficiency patients (3)
- ☐ False positive urine drug test (4)

End of Block: Block 2

Start of Block: Block 3

Q14 Do you have any concepts you hope are addressed in these cultural complications discussions?

____

Q15 Do you have any additional comments or suggestions on the structure/content of the UC Davis Cultural Complications Curriculum?

____

End of Block: Block 3
