# Supplementary material for: Integration of a Cultural Complications Curriculum Into a Surgery Department Conference
Source: JAMA Netw Open. 2025 Jun 27;8(6):e2517811. doi: 10.1001/jamanetworkopen.2025.17811 (PMC12205394; doi:10.1001/jamanetworkopen.2025.17811)
Supplement: Supplement 2. — Data Sharing Statement [file jamanetwopen-e2517811-s002.pdf]

Data Sharing Statement

Fannon. Integration of a Cultural Complications Curriculum Into a Surgery Department Conference. *JAMA Netw Open*. Published June 27, 2025.
doi:10.1001/jamanetworkopen.2025.17811

Data
Data available: Yes
Data types: Deidentified participant data
How to access data: efannon99@gmail.com
When available: With publication

Supporting Documents
Document types: None

Additional Information
Who can access the data: researchers whose proposed use of the data has been approved
Types of analyses: any
Mechanisms of data availability: after approval of a proposal
